# Transvaginal bowel evisceration and gangrenous small-bowel obstruction after self-amputation of neglected uterovaginal prolapse: a case report and review of literature

**DOI:** 10.1093/jscr/rjaf852

**Published:** 2025-10-24

**Authors:** Dereje Gashaw Andargie, Biruk T Mengistie, Chernet T Mengistie, Yonas T Habtemariam, Eyaya Misgan Asres, William Rutagengwa, Temesgen Tantu, Amanuel Sisay Endeshaw

**Affiliations:** Department of Surgery, College of Medicine and Health Sciences, Bahir Dar University, Peda (Kebele 08), PO Box 79, Bahir Dar, Amhara Region, Ethiopia; School of Medicine, College of Health Sciences, Addis Ababa University, Entoto Road, PO Box 9086, Addis Ababa, Ethiopia; School of Medicine, College of Health Sciences, Addis Ababa University, Entoto Road, PO Box 9086, Addis Ababa, Ethiopia; Department of Surgery, College of Medicine and Health Sciences, Bahir Dar University, Peda (Kebele 08), PO Box 79, Bahir Dar, Amhara Region, Ethiopia; Department of Obstetrics and Gynecology, College of Medicine and Health Sciences, University of Rwanda, KG 11 Avenue (Gasabo/Remera area), PO Box 3286, Kigali, Rwanda; Partners In Health (Inshuti Mu Buzima), Kirehe Program, Kirehe District Hospital, PO Box 45, Kibungo, Kirehe District, Eastern Province, Rwanda; Department of Obstetrics and Gynecology, College of Medicine and Health Sciences, University of Rwanda, KG 11 Avenue (Gasabo/Remera area), PO Box 3286, Kigali, Rwanda; Department of Anesthesia, College of Medicine and Health Sciences, Bahir Dar University, Peda (Kebele 08), PO Box 79, Bahir Dar, Amhara Region, Ethiopia

**Keywords:** small bowel obstruction, gangrenous, uterovaginal prolapse, self-attempted cutting

## Abstract

Neglected uterovaginal prolapse (UVP) can lead to life-threatening complications. We report a 45-year-old para 5 woman with a 10-year history of UVP who presented after 2 days of a protruding vaginal mass, bilious vomiting, fever, and abdominal pain following an attempt to cut the prolapsed tissue with a blade. On examination, she was tachycardic, hypotensive, actively bleeding from the prolapse, and showed signs of small-bowel evisceration. Emergency laparotomy identified a 4-cm anterior uterine defect with 60 cm gangrenous ileum herniated ~15 cm from the ileocecal junction; the necrotic segment was resected and an ileostomy created, and definitive repair of the prolapse was performed. The patient recovered and was discharged on postoperative day seven. This case shows that neglected UVP may prompt desperate, dangerous self-interventions and can cause transvaginal bowel evisceration and strangulation; early recognition, prompt multidisciplinary surgical care, and attention to psychosocial barriers to care are essential to prevent such outcomes.

## Introduction

Uterovaginal prolapse (UVP) is a form of pelvic organ prolapse (POP) in which one or more pelvic organs descend into or through the vaginal canal due to weakness of the pelvic support structures [1]. POP is a common disorder; a recent meta-analysis reported an overall prevalence of about 30% in women examined clinically [1]. Prolapse is especially prevalent in multiparous and older women, with some studies estimating that up to 50% of women over age 50 have some degree of prolapse [2]. Recognized risk factors include high parity, advancing age, obesity, chronic straining (such as from constipation), and occupations involving heavy lifting [1, 2].

Neglected or advanced prolapse can profoundly impair a woman’s health and well-being. Affected women often experience lower urinary tract symptoms (such as stress or urge incontinence, frequency, or retention) as well as pelvic symptoms (vaginal heaviness, discharge, bleeding, or constipation) [3]. Moreover, POP frequently leads to sexual dysfunction and psychological distress: patients commonly report dyspareunia, embarrassment, anxiety, and loss of self-esteem [2, 3]. These physical and emotional burdens can result in social stigma and isolation; for example, women with longstanding prolapse in some communities have suffered domestic repercussions due to shame [1, 2].

Small bowel obstruction (SBO) is a common surgical emergency that accounts for roughly 15% of gastrointestinal surgical admissions in the United States (~350 000 cases per year) [4]. The leading causes of SBO include postoperative adhesions (from prior surgeries), intra-abdominal malignancy, Crohn’s disease, and hernias. In one series, adhesions accounted for about 74% of SBO cases [5]. Gynecologic and obstetric procedures, for example, hysterectomy, are well-known sources of pelvic adhesions and subsequent SBO; in fact, one study found that SBO occurred in about 1.6% of patients after abdominal hysterectomy (16.3 per 1000 procedures), whereas the incidence after cesarean delivery was much lower (≈0.05%) [6].

In contrast, SBO due to direct transvaginal evisceration of bowel through a prolapsed uterus is exceedingly rare. Published reports of vaginal evisceration typically involve elderly women with long-standing prolapse, often precipitated by sudden straining or trauma [7]. Such events are recognized as surgical emergencies due to the risk of rapid bowel ischemia. In our case, however, the evisceration and resulting gangrenous SBO were caused by a unique mechanism: the patient’s self-attempted amputation of the prolapsed uterus. To our knowledge, SBO caused by deliberate self-amputation of a prolapsed uterus has not been previously described.

## Case presentation

**Figure 1:**
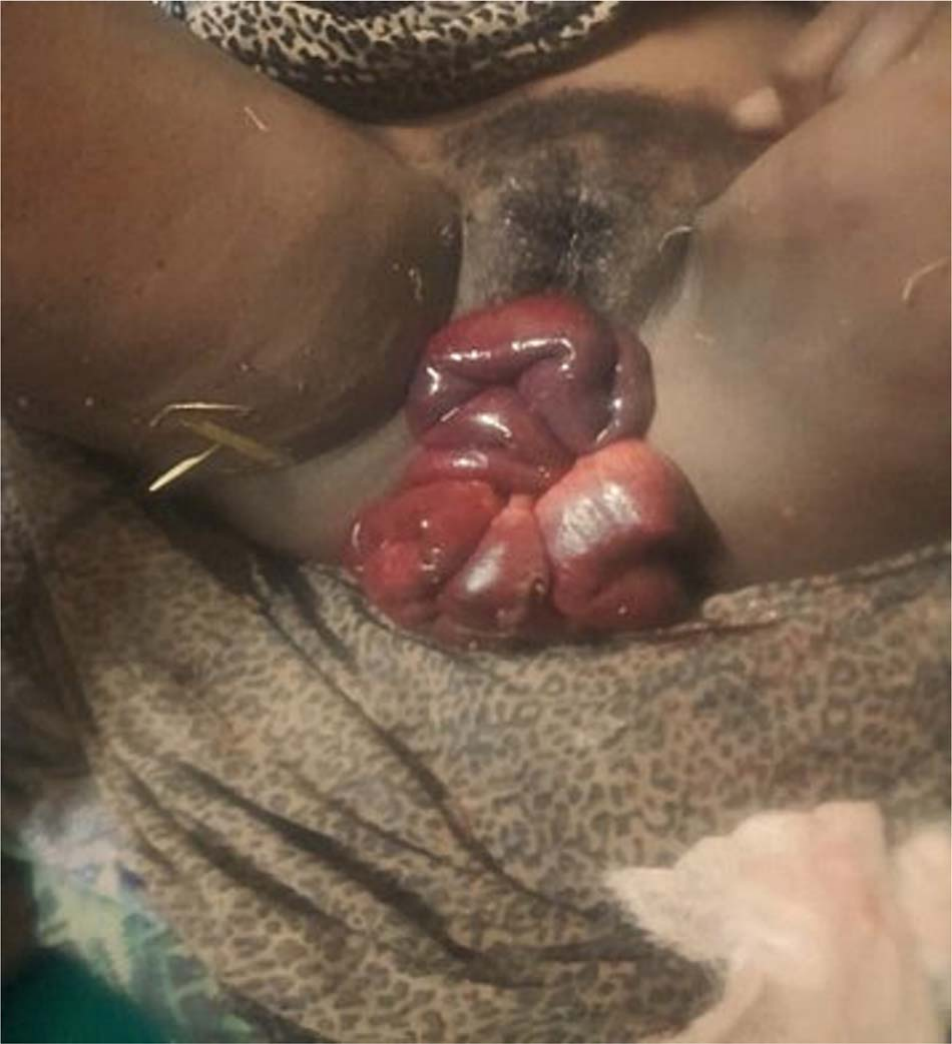
Complete herniation of gangrenous small bowel eviscerated through the self-amputated UVP.

A 45-year-old, para-5 woman presented with a protruding vaginal mass for 2 days that was associated with bilious vomiting, fever, and abdominal pain. She reported a long-standing mass per vagina of 10 years duration. She attempted to cut the prolapsed mass with a local blade 3 days before the current presentation. The pain was intermittent, crampy initially, which later became continuous and severe on the second day. She denied any history of abdominal surgery, history of trauma, recent weight loss, or bowel habit change until the start of the current complaint. Examination revealed tachycardia, fever, hypotension, active bleeding from the protruding mass per vagina, and a gangrenous herniated small bowel. We made a diagnosis of gangrenous SBO and started symptomatic treatment with fasting, aggressive fluid replacement, gastrointestinal decompression, and antibiotics, followed by emergency exploratory laparotomy (Fig. 1).

**Figure 2:**
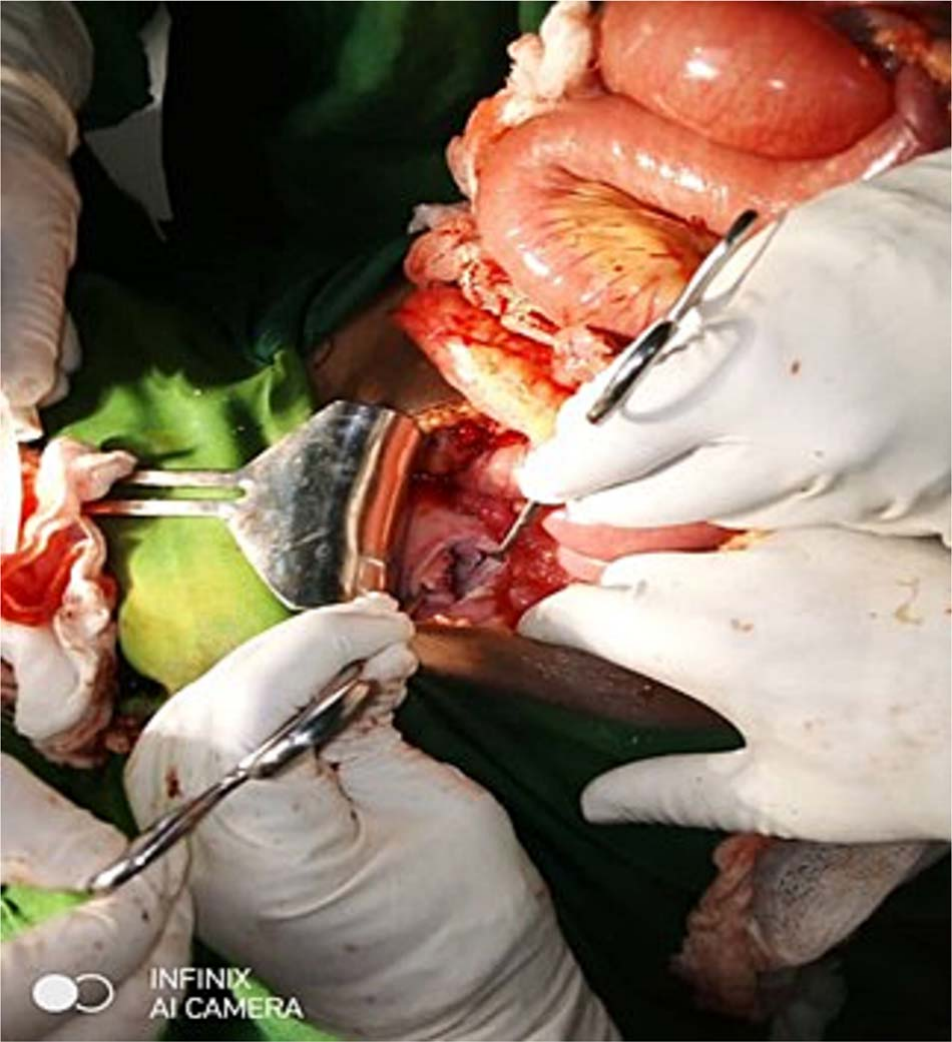
Anterior lower uterine body defect resulted from the self-attempted amputation of UVP.

An exploratory laparotomy was performed with a team of surgeons and gynecologists. Intraoperatively, we found about 10–12 weeks size nongravid uterus with a 4 cm anterior uterine defect around the lower uterine body and a 60 cm gangrenous herniated small bowel about 15 cm proximal to the ileocecal junction. We resected the gangrenous segment of the ileum and created a loop ileostomy. We did a total abdominal hysterectomy with vaginal vault suspension for the prolapsed uterus. The patient was successfully discharged with a functional ileostomy on postoperative day seven (Fig. 2).

## Discussion

SBO remains one of the most common emergency surgical admissions. In adults, postoperative adhesions are the leading cause of SBO [5, 8]; Other causes include hernias, malignancy, and Crohn’s disease [5]. Gynecologic and obstetric surgeries contribute to SBO risk by causing adhesions. For example, a large series reported SBO in about 1.6% of women after abdominal hysterectomy (16.3 per 1000 operations), whereas the rate after cesarean section was only ≈0.05% [6]. Pregnancy termination with instrumentation can also perforate the uterus; if unrecognized, this may allow bowel injury or prolapse into the uterine cavity, but SBO from such events is exceedingly rare [9].

In our patient, the mechanism of SBO differed entirely from the usual adhesive process. The patient’s attempted incision in the prolapsed uterus created a defect in the uterine wall and vaginal vault. Through this defect, a loop of the ileum herniated and became strangulated. The narrow aperture rapidly compromised the bowel’s blood supply, resulting in gangrene. Such a mechanism produces rapid vascular compromise because the small, rigid defect behaves as a strangulating orifice; case reports and series of transvaginal evisceration demonstrate that bowel ischemia and necrosis can ensue within hours, markedly increasing the risk of sepsis and death if operative repair is delayed [7, 10]. Initial management, therefore, focuses on protection of exposed bowel (moist sterile packing), aggressive resuscitation and broad-spectrum antimicrobials, and expedited operative exploration; the operative approach (transvaginal, transabdominal, or combined) should be individualized according to bowel viability and the character of the pelvic defect [7, 11]. Multidisciplinary involvement of both general surgeons and gynecologic surgeons improves the ability to restore gastrointestinal continuity and definitively repair pelvic floor defects, and recent reports highlight better outcomes when coordinated strategies are employed for complex cases of vaginal evisceration [4].

Most reported cases of vaginal evisceration have occurred in older women with known uterine or vault prolapse [7]. Our case is distinguished by the self-inflicted nature of the injury. No trained professional was involved; rather, the patient’s desperate attempt to “cut” the prolapse (believing it was incurable) led to life-threatening bowel injury. The occurrence of SBO from such an etiology has not, to our knowledge, been documented in the literature. This case underscores how neglected UVP can lead to unusual and severe complications outside the typical clinical scenarios. When evaluating prolapse patients, clinicians should be aware that neglected disease may present with unexpected emergencies, and a thorough history, including psychosocial factors, may help identify individuals at risk.

## Conclusion

This case highlights the profound adverse impact that untreated UVP can have on women’s health and well-being. POP is recognized as a major cause of morbidity and disability in women, substantially impairing physical function and quality of life. Advanced prolapse can force women to endure years of pain, discomfort, and social embarrassment. Awareness of the heavy psychosocial burden of prolapse is therefore important: clinicians should elicit a careful history of pelvic symptoms and their effects on daily life so that neglected cases can be recognized and treated before life-threatening complications arise.

## Data Availability

Data sharing is not applicable to this article as no datasets were generated or analyzed during the current study.
